# Blood Unit Discard Rates Due to Transfusion–Transmissible Infections and Non‐Infectious Causes and Their Associated Factors in Eastern and Northeastern Parts of Amhara Regional State, Ethiopia: A Retrospective Cross‐Sectional Study From 2022–2025

**DOI:** 10.1002/hsr2.73354

**Published:** 2026-10-07

**Authors:** Tesfaye Gessese, Afewerk Habtamu Aseme, Zewudu Mulatie, Mikiyas Shemelis Achame, Sisay Desale, Saleamlak Sebsibe, Yonas Erkihun, Yeshimebet Kassa, Mahider Shimelis Feyisa, Mihreteab Alebachew, Bruktawit Eshetu, Tadesse Sisay

**Affiliations:** ^1^ Department of Medical Laboratory Sciences, College of Medicine and Health Sciences Wollo University Dessie Ethiopia; ^2^ School of Medical Laboratory Sciences, Asrat Weldeyes Health Science Campus Debre Berhan University Debre Berhan Ethiopia; ^3^ Dessie Hospital Dessie Dessie Ethiopia

**Keywords:** blood bank, blood discard, transfusion‐transmissible infections and non‐transfusion‐transmissible infections

## Abstract

**Background:**

Blood is an irreplaceable medical resource, yet blood unit discard remains a significant challenge for transfusion services. This study aimed to evaluate discard rates by integrating both infectious (TTI) and non‐infectious causes and identifying factors associated with blood wastage to provide evidence to support strategies for improving blood safety.

**Methods:**

An institutional‐based retrospective cross‐sectional study was conducted using data from 2022 to 2025 across Dessie, Kemise, Woldia, and Sekota Blood Banks. Data were extracted from Blood Bank databases using a structured checklist and analyzed with SPSS version 26.0. Both univariable and Multivariable logistic regression were performed to identify predictors of transfusion transmissible infections (TTIs) and non‐infectious causes, with statistical significance set at *p* < 0.05.

**Results:**

Records from 60,008 donors and 67,976 blood products were reviewed. A total of 6,130 units (9.02%) were discarded. TTIs accounted for 4.6% of discards, led by Hepatitis B virus (2.0%) and Syphilis (1.2%). Non‐infectious causes contributed 4.4%, primarily due to expiration (2.0%). ABO group, Rh factor, donor age, donor type, collection site, and Blood Bank location were identified as significant predictors of discard.

**Conclusion:**

The study reveals a notable blood unit discard rate of 9.02%, driven equally by infectious transfusion‐transmissible infections and non‐infectious factors like expiration. Key risk factors include donor demographics, blood group, and specific blood bank locations, highlighting the need for better inventory management and targeted donor selection.

## 1 Introduction

### 1.1 Background

Blood transfusion is a key fundamental component in modern health care in saving the lives of many people in routine and emergency situations, often being the only lifesaving option in cases like severe hemorrhage, obstetric emergencies, trauma, major surgery, chronic anemia, and surgical procedures [1, 2]. So far donated blood is a scarce and precious resource; ensuring a safe and sufficient blood supply is a key public‐health priority to protect recipients from serious and unwanted risks. As a result, World Health Organization (WHO) recommend a well‐organized blood transfusion service, with quality systems in all areas; when blood fail those quality system checks or are compromised otherwise, they must be discarded, WHO recognize both infectious marker reactivity and quality issues (like, incomplete collection, storage problems, hemolysis) as common reasons for blood discard. Studies across different countries confirm that discard of blood and its components is a substantial and persistent problem despite efforts to recruit donors and supply safe blood [3].

The major transfusion‐transmissible infections (TTIs), are Hepatitis B virus (HBV), Hepatitis C virus (HCV), Human immunodeficiency virus (HIV), and Syphilis (Treponema pallidum) screening those infections are the minimum recommendation worldwide [4]. TTI sero‐reactivity leading, non‐negotiable cause of discard globally accounts for a substantial share of all discarded units. For example, In an Indian study on both whole blood and its components, sero‐positivity accounted for approximately 8.6% of overall blood wastage [5]. in a multi‐center review at a tertiary hospital in Ghana, TTI positivity accounted for 54.9% of discarded units [6]. In Ethiopia a systematic review and meta‐analysis showed that the pooled sero‐epidemiology of HBV, HIV, HCV, and syphilis among blood donors was 5.20%, 2.83%, 0.93%, and 1.50%, respectively.

Another nationwide retrospective study of 554,954 donors across 14 Blood Bank facilities in Ethiopia (2014–2019) documented sero‐prevalence rates of 2.4% for HBV, 0.4% for HCV, 0.4% for HIV, and 0.9% for syphilis. The combined TTI positivity rate ranged from 5.7% in 2014 down to 3.4% in 2019 [7].

Apart from infectious risks, non‐infectious factors such as expiration, hemolysis, storage problems, leakage, lipemia, clotting, underweight or overweight blood collection, or other quality/handling issues may also render donated blood units unsuitable for transfusion, leading to discard. However, it is remains under‐reported in many low‐income countries. A study in Zalawad region of Saurashtra, India, revealing that technical issues (clots, improper collection) and storage expiry are key reasons, aiming to help improve blood management by reducing wastage through better donor screening, component preparation, and stock rotation, a common challenge in Blood Banking [8]. Another study from India the average discard rate for packed red cell (CRC), fresh frozen plasma (FFP), platelet concentrate (PC), and cryoprecipitate was 4.95%, 2.46%, 19.12%, and 3.03%, respectively. The most common reason for CRC and PC discard was expiry followed by positivity TTIs, whereas discard of FFPs was due to breakage of the component [9]. In Ethiopia, extant research predominantly focuses on TTI prevalence among donors. However, to the best of our knowledge there is no published comprehensive study in Ethiopia that systematically quantifies the discard rates of donated blood units considering both infectious (TTI) and non ‐infectious causes in a unified analysis. This gap limits a complete understanding of blood wastage and the inefficiencies in the blood supply system in Ethiopia. Therefore, this study aimed to determine the prevalence of blood unit discard due to both transfusion ‐transmissible infections (TTIs) and non‐transfusion‐transmissible (non‐TTI) causes and to assess factors associated with blood unit discard among blood donors in four Blood Banks in northeastern Ethiopia using a retrospective cross‐sectional study design. The findings are expected to provide evidence to support strategies for improving blood safety, optimizing inventory management, reducing blood wastage, and strengthening Blood Bank services in Ethiopia.

## 2 Methods

### 2.1 Study Setting and Period

The study was carried out at four Blood Banks: Dessie, Kemise, Woldia and Kemise Blood Bank facilities in, Eastern and Northeastern parts of Amhara Regional State, Ethiopia over a defined study period (from January 2022 to June 2025). These Blood Banks provide blood collection, screening, storage, and distribution services to public and private health facilities. All four facilities operate under the Ethiopian Blood and Tissue Bank Service (EBTBS). Together, they form a network that supplies blood to surrounding public and private health facilities.

### 2.2 Study Design

An institutional‐based retrospective study was conducted.

### 2.3 Population

The source population consists of all blood donor records registered at Dessie, Kemise, Woldia and Kemise Blood Bank facilities in East Amhara, northeast Ethiopia, while the study population consists of all blood donor records registered at Dessie, Kemise, Woldia and Kemise Blood Bank facilities in East Amhara, northeast Ethiopia during the study period.

### 2.4 Inclusion and Exclusion Criteria

**Inclusion Criteria:** All collected blood units recorded in the Blood Bank database and/or discard registers during the study period.

**Exclusion Criteria:** Records with incomplete or missing information was excluded.

### 2.5 Data Collection Procedure

Data were collected retrospectively from Blood Bank registers, screening logs, and electronic information systems using a structured data extraction checklist. The checklist included donor socio ‐demographic characteristics (age, sex, and donation type), blood group, donation frequency, screening results for TTIs, and reasons for blood unit discard. TTIs screened routinely included HIV, HBV, HCV, and syphilis, using Chemiluminescence Immunoassay technology by Alinity ‐ I machine, following national blood transfusion guidelines. Blood component preparation (CRC, FFP, and PLT) was conducted via the manual centrifuge method at the Dessie Blood Bank. Non ‐ infectious causes of discard such as insufficient volume, hemolysis, clotting, leakage, expiration, and storage ‐related issues were also recorded in laboratory logbooks. Since in Ethiopia whole blood is routinely used, whole blood collection was conducted across all four Blood Bank sites, blood component preparation (CRC, FFP, and PLT) was performed exclusively at Dessie Blood Bank laboratory according to standard national protocols. Therefore, the analyses regarding component discard rates specifically reflect the operational outcomes of this single facility.

### 2.6 Study Variables

We have extracted information on donor demographic characteristics, including age, sex, donor type (voluntary or family/replacement), site of donation (mobile or static), Blood Bank, year of donation, blood group (ABO and Rh). Information on blood unit outcomes was also collected, including discard status (discarded or not discarded), reason for discard, (TTI ‐related or non ‐TTI ‐related). For component analyses, data on component type (whole blood, CRC, FFP, and PLT) were collected.

### 2.7 Data Quality Assurance

To ensure data quality, the data extraction tool was pretested prior to the actual data collection. Data collectors were trained on the study objectives and data collection procedures. Quality control for Alinity ‐I machine was maintained through daily calibration and the inclusion of known positive and negative controls in every run to ensure analytical validity. All procedures followed the Ethiopian Blood Bank Service Standard Operating Procedures. To ensure the accuracy of non ‐TTI data, the Blood Bank facilities follow a strict SOP where every unit discarded for non ‐infectious reasons must be documented in a dedicated logbook with the specific reason for rejection. laboratory logbooks are maintained daily by laboratory personnel and are subjected to monthly auditing and reporting to the relevant governing bodies. Collected data were checked daily for completeness and consistency. Any missing or inconsistent data were cross ‐verified with source documents where possible.

### 2.8 Data Processing and Analysis

Data were entered into and analyzed using Statistical Package for the Social Sciences (SPSS) version 26.0 statistical software. Descriptive statistics such as frequency and proportions were calculated and presented via text and tables. Bivariable and multivariable logistic regression analyses were performed to identify factors associated with blood unit discard. To account for potential confounders, variables having a *p*‐value < 0.25 in the bivariable analysis were added to the multivariable logistic regression model. The Variance Inflation Factor (VIF < 10) and tolerance parameters were used to examine multicollinearity among independent variables. The Hosmer–Lemeshow goodness‐of‐fit test was used to evaluate the model's fitness. The 95% confidence intervals (CIs) for the crude odds ratios (COR) and adjusted odds ratios (AOR) were calculated. A two‐tailed *p*‐value < 0.05 was considered statistically significant.

### 2.9 Ethical Consideration

Ethical approval was obtained from the Wollo University Ethical Review Board. Permission to access Blood Bank records was secured from the respective authorities. Informed patient/participant consent was formally waived by the IRB due to the completely retrospective and anonymized nature of the secondary aggregate data. Confidentiality was maintained by excluding personal identifiers and using the data solely for research purposes.

## 3 Results

### 3.1 Socio Demographic and Donation Characteristics of the Study Participants

A total of 60,008 blood donors and 67,976 blood units over a 4 year study period (2022–2025) from Dessie, Kemise, Woldia, Sekota Blood Banks were included. Most blood was obtained from volunteer blood donors 99.7% (59,828/60,008), and 85.0% (51,007/60,008) of all donations were collected by mobile sites. The majority of donors were male 63.7% (38,206/60,008). The median age was 26 with inter‐quartile ranges (IQR) of 14.9 years with the majority aged between 18 and 27, 61% (36,624/60,008). The majority of blood units 82.9%, (56,024/67,976) were whole blood, while component preparation was practiced for 5.9% (3,984/67,976) of units, yielding 11,952 specific components (CRC, FFP, and PLT; 3,984 units each) (Table 1).

**Table 1:** Socio‐demographic profile, donation characteristics and blood groups distribution (*n* = 60,008) of blood donors in eastern and northeastern parts of the Amhara Regional State, Ethiopia.

Variable	Category	Frequency (*n*)	Percentage (%)
Sex	Male	38,206	63.7
	Female	21,802	36.3
Age group	18–27	36624	61
	28–37	11000	18.3
	≥ 38	12384	20.6
Donor type	Volunteer	59,809	99.7
	Family/Replacement	199	0.3
Collection site	Mobile	51,020	85.0
	Center (Static)	8,988	15.0
Component prepared	Yes	3984	6.6
	No	56024	93.4
Name of blood bank	Dessie	38428	64.0
	kemise	4647	7.7
	Woldia	12724	21.2
	Sekota	4209	7.0
Year	2022	16879	28.1
	2023	17283	28.8
	2024	16361	27.3
	2025	9485	15.8
ABO group	O	21,024	35
	A	19,987	33.3
	B	15,404	25.7
	AB	3,593	6
Rh factor	Rh Positive	53,846	89.7
	Rh Negative	6,162	10.3

Dessie Blood Bank contributed to 64.0% (38,428/60,008) of all donations, while Sekota Blood Bank contributed the least 7% (4,209/60,008). Blood group O was the most common 35.0% (21,024/60,008), and AB was 6.0% (3,593/60,008). The majority of blood blood units were Rh positive 89.7% (53,846/60,008) (Table 1).

### 3.2 Magnitude of Blood and Blood Component Discards

From a total of 67,976 blood products (including whole blood and prepared components), the overall discard rate was 9.02% (6,130/67,976) (95% CI: 8.80%–9.23%). From the total discard 8.01% (5,444/67,976) were whole blood units (Tables 2) and 1.01% (686/67,976) was blood components (Table 3). PLT discard rate was 0.5% (367/67,976) followed by FFP 0.4% (242/67,976) and CRC 0.1% (77/67,976). From a total of 11952 prepared blood component, the overall discard rate across all components was 5.7% (686/11,952), from this PLT discard rate was the most common 3.1% (367/11,952) (Table 3). Dessie Blood Bank accounted for the largest overall discard proportion at 5.8% (3953/67,976), while Sekota Blood Bank was the least 0.7% (500/67,976). However, across Blood Banks, Kemise Blood Bank was 12% (557/4647) discard proportion followed by Sekota 11.9% (500/4,209), Woldia 8.8% (1,120/12,724), and Dessie 8.5% (3,953/46,396). The combined regional discards follow an overall increasing trend, moving from 8.9% (1,691/18,973) in 2022 to a peak of 10.1% (1,083/10,674) in 2025.

**Table 2:** Magnitude of whole blood discard due to TTIs and non‐TTIs causes by Year and Blood Bank in Eastern and Northeastern parts of the Amhara Regional State, Ethiopia (*n* = 56024).

Name	Cause of discard	2022	2023	2024	2025	Total
Dessie	HBV	186 (2,0%)	173 (1.6%)	206 (2.4%)	165 (2.8%)	730 (2.1%)
	Syphilis	161 (1.7%)	100 (0.9%)	82 (1.0%)	73 (1.2%)	416 (1.2%)
	HIV	91 (1.0%)	115 (1.1%)	108 (1.3%)	55 (0.9%)	369 (1.1%)
	HCV	66 (0.7%)	49 (0.5%)	52 (0.6%)	46 (0.8%)	213 (0.6%)
	Multiple	6 (< 0.1%)	7 (< 0.1%)	8 (< 0.1%)	2 (< 0.1%)	23 (< 0.1%)
	Expired	187 (2.0%)	129 (1.2%)	97 (1.1%)	236 (4.0%)	649 (2.4%)
	Discoloration	13 (0.1%)	3 (< 0.1%)	10 (0.1%)	55 (0.9%)	81 (0.3%)
	Hemolysis	19 (0.2%)	5 (< 0.1%)	6 (< 0.1%)	0 (0.0%)	30 (< 0.1%)
	Clotting	6 (< 0.1%)	6 (< 0.1%)	13 (0.1%)	0 (0.0%)	25 (< 0.1%)
	Small	199 (2.1%)	186 (1.7%)	187 (2.2%)	65 (1.1%)	637 (1.4%)
	Overweight	10 (0.1%)	9 (< 0.1%)	10 (0.1%)	6 (0.1%)	35 (< 0.1%)
	wrong temperature	15 (0.2%)	24 (0.2%)	25 (0.3%)	0 (0%)	64 (0.1%)
	Expired	187 (2.0%)	129 (1.2%)	97 (1.1%)	236 (4.0%)	649 (2.4%)
	Total discard	959 (10.3%)	801 (7.5%)	804 (9.5%)	703 (11.8%)	3267 (9.5%)
	Total collection	9333	10667	8496	5948	34444
Kemise	HBV	8 (0.8%)	56 (3.7%)	24 (2.2%)	8 (0.8%)	96 (1.9%)
	Syphilis	2 (0.2%)	42 (2.8%)	47 (4.2%)	17 (1.7%)	108 (2.1%)
	HIV	2 (0.2%)	10 (0.7%)	16 (1.4%)	1 (0.1%)	29 (0.4%)
	HCV	2 (0.2%)	10 (0.7%)	10 (0.9%)	5 (0.5%)	27 (0.6%)
	Multiple	2 (0.2%)	2 (0.1%)	3 (0.3%)	1 (< 0.1%)	8 (0.2%)
	Expired	11 (1.1%)	13 (0.8%)	33 (3%)	16 (1.6%)	73 (1.6%)
	Small	22 (2.2%)	38 (2.5%)	34 (3.1%)	16 (1.6%)	110 (2.4%)
	overweight	16 (1.6%)	18 (1.2%)	6 (0.5%)	5 (0.5%)	45 (0.9%)
	Other	12 (1.2%)	22 (1.5%)	21 (1.9%)	6 (0.6%)	61 (1.3%)
	Total discard	77 (7.7%)	211 (13.9%)	194 (17.4%)	75 (7.4%)	557 (12%)
	Total collection	1004	1516	1114	1013	4647
Woldia	HBV	105 (2.4%)	67 (2.1%)	87 (2.4%)	28 (1.8%)	287 (2.3%)
	Syphilis	85 (1.9%)	37 (1.2%)	54 (1.5%)	17 (1.1%)	193 (1.5%)
	HIV	52 (1.2%)	18 (0.6%)	27 (0.7%)	6 (0.4%)	103 (0.8%)
	HCV	47 (1.1%)	18 (0.6%)	35 (1.0%)	6 (0.4%)	106 (0.8%)
	Multiple	3 (< 0.1%)	6 (0.2%)	7 (0.2%)	4 (0.3%)	20 (0.2%)
	Expired	19 (0.4%)	26 (0.8%)	28 (0.8%)	29 (1.8%)	102 (0.8%)
	Discoloration	23 (0.5%)	16 (0.5%)	18 (0.5%)	17 (1.1%)	74 (0.6%)
	Small weight	49 (1.1%)	35 (1.1%)	35 (0.9%)	24 (1.5%)	143 (1.1%)
	Over weight	11 (0.3%)	8 (0.3%)	23 (0.6%)	11 (0.7%)	53 (0.4%)
	Other	8 (0.2%)	8 (0.3%)	10 (0.3%)	13 (0.8%)	39 (0.3%)
	Total discard	402 (9.1%)	239 (7.7%)	324 (8.9%)	155 (9.8%)	1120 (8.8%)
	Total collection	4394	3124	3620	1586	12724
Sekota	HBV	41 (2.8%)	54 (5.2%)	53 (4.8%)	24 (3.7%)	172 (4.1%)
	Syphilis	5 (0.3%)	14 (1.4%)	24 (2.2%)	9 (1.4%)	52 (1.2%)
	HIV	6 (0.4%)	8 (0.8%)	6 (0.5%)	7 (1.1%)	27 (0.6%)
	HCV	3 (0.2%)	11 (1.1%)	8 (0.7%)	4 (0.6%)	26 (0.6%)
	Multiple	3 (0.2%)	2 (0.2%)	2 (0.2%)	1 (0.2%)	8 (0.2%)
	Expired	19 (1.3%)	19 (1.8%)	28 (2.5%)	13 (2%)	79 (1.9%)
	Small weight	24 (1.7%)	20 (1.9%)	23 (2.1%)	16 (2.5%)	83 (2%)
	Other	7 (0.5%)	16 (1.2%)	21 (1.9%)	9 (1.4%)	53 (1.2%)
	Total discard	108 (7.8%)	144 (14.0%)	165 (15.0%)	83 (13.9%)a	500 (11.9%)
	Total collection	1440	1030	1097	642	4209
Grand total	Discard	1546 (9.6%)	1393 (8.5%)	1484 (10.4%)	1016 (11.1%)	5444 (9.7%)
	Collection	16171	16337	14327	9189	56024

**Table 3:** Component discard (3984 each, total 11952) in Dessie blood bank Northeastern parts of the Amhara Regional State, Ethiopia.

Blood componenet	Reason	2022	2023	2024	2025	Total
CRC	HBV	4 (0%)	6 (0.5%)	7 (0.5%)	2 (0.4%)	19 (0.5%)
	Syphilis	0 (0%)	5 (0.4%)	7 (0.5%)	3 (0.6%)	15 (0.4%)
	HIV	0 (0%)	0 (0%)	4 (0.3%)	1 (0.2%)	5 (0.1%)
	Expired	1 (0.1%)	0 (0%)	0 (0%)	3 (0.6%)	4 (0.1%)
	Leakage	0 (0%)	0 (0%)	0 (0%)	0 (0%)	0 (0%)
	Discoloration	3 (0.3%)	14 (1.3%)	10 (0.7%)	3 (0.6%)	30 (0.7%)
	Small	0 (0%)	0 (0%)	2 (0.1%)	2 (0.4%)	4 (0.1%)
Total discard		8 (0.8%)	25 (2.2%)	30 (2.1%)	14 (2.8%)	77 (1.9%)
Total collection		934	1120	1435	495	3984
FFP	HBV	4 (0.4%)	6 (0.5%)	7 (0.5%)	2 (0.4%)	19 (0.5%)
	Syphilis	0 (0%)	5 (0.4%)	7 (0.5%)	3 (0.6%)	15 (0.4%)
	HIV	0 (0%)	0 (0%)	4 (0.3%)	1 (0.2%)	5 (0.1%)
	Expired	40 (4.3%)	65 (5.8%)	80 (5.6%)	15 (3%)	200 (5%)
	Leakage	3 (0.3%)	0 (0%)	0 (0%)	0 (0%)	3 (0.1%)
	Discoloration	0 (0%)	0 (0%)	0 (0%)	0 (0%)	0 (0%)
	Small	0 (0%)	0 (0%)	0 (0%)	0 (0%)	0 (0%)
Total discard		47 (5%)	76 (6.8%)	98 (6.8%)	21 (4.2%)	242 (6.1%)
Total collection		934	1120	1435	495	3984
PLT	HBV	4 (0%)	6 (0.5)	7 (0.5%)	2 (0.4%)	19 (0.5%)
	Syphilis	0 (0%)	5 (0.4%)	7 (0.5%)	3 (0.6%)	15 (0.4%)
	HIV	0 (0%)	0 (0%)	4 (0.3%)	1 (0.2%)	5 (0.1%)
	Expired	69	79 (7.1%)	100 (6.9%)	21 (4.2%)	269 (6.8%)
	leakage	3 (0.3%)	7 (0.6%)	3 (0.2%)	0 (0%)	13 (0.3%)
	Discoloration	4 (0.4%)	4 (0.4%)	6 (0.4%)	2 (0.4%)	16 (0.4%)
	Small	10 91%	13 (1.2%)	4 (0.3%)	3 (0.6%)	30 (0.8%)
Total discard		90 (9.6%)	114 (10.2%)	131 (9.1%)	32 (6.5%)	367 (9.2%)
Total collection/processed		934	1120	1435	495	3984

Abbreviations: CRC, concentrated red cell; FFP, fresh frozen plasma, PLT, platelet.

#### 3.2.1 Discard Due to TTIs

TTIs cause accounts for 4.6% (3,130/67,976) discard rate (3013 whole blood and 117 blood component). The most prevalent infectious cause was HBV 2% (1342/67,976), followed by Syphilis 1.2% (814/67,976). Across Blood Banks, Sekota was the highest 6.8% (285/4,209), followed by Kemise 5.8% (268/4,647), Woldia 5.6% (709/12,724), and Dessie 4% (1,868/46,396). Regional TTI discard rates were 4.7% (888/18,747) in 2022, 4.3% (832/19,523) in 2023, 4.7% (913/19,231) in 2024% and 4.7% (497/10,475) in 2025.

#### 3.2.2 Discard Due to Non ‐TTIs Causes

The overall discard rate of non ‐TTIs cases was 4.4% (3,000/67,976) (2,431 whole blood and 569 component). Expiry accounted for 2% (1,376/67,976) (903 whole blood + 473 component units) of the total collections, and underweight units 1.5% (1,008/67,976) (974 whole blood + 34 for blood component units). Dessie Blood Bank recorded a non ‐TTI discard rate of 4.5% (2,085/46,396) (whole blood 1,516 + components 569). In Kemise, Woldia, and Sekota Blood Banks non ‐TTI discard rate were 6.2% (289/4647), 3.2% (411/12724), and 5.1% (215/4209) respectively. In terms of the study period 4.2% (803/18,973) in 2022, 3.9% (778/19,697) in 2023, 4.5% (833/18,632) in 2024, and 5.5% (586/10,674) in 2025.

### 3.3 Associated Factors

#### 3.3.1 Factors Associated With TTI Related Blood Unit Discard

Bivariable and multivariable analysis were conducted to identify associated factors for both TTI and non‐TTI related blood unit discard. Donor type (family/replacement), donor age (28–37 and ≥ 38), and Blood Bank site (Kemise, Woldia, Sekota) and blood group (group AB) were the associated factors of TTI‐related blood unit discard. Compared with the aged 18–27 years, donors aged 28–37 and aged ≥ 38 years had significantly higher odds of blood unit discard (AOR = 2.33, 95% CI 2.13–2.55; *p *< 0.001) and (AOR = 2.38, 95% CI 2.18–2.60; *p *< 0.001) respectively. Similarly, family (replacement) donors showed higher odds of discard compared with volunteer donors (AOR = 3.01, 95% CI 2.06–4.41; *p *< 0.001). Regarding Blood Bank location, Kemise (AOR = 1.47, 95% CI 1.29–1.68; *p *< 0.001), Woldia (AOR = 1.38, 95% CI 1.26–1.51; *p *< 0.001), and Sekota (AOR = 1.73, 95% CI 1.52–1.96; *p *< 0.001) had significantly higher odds of discard as compared with Dessie Blood Bank (Table 4).

**Table 4:** Logistic regression for factors associated with TTI related blood unit discard in Eastern and Northeastern parts of the Amhara Regional State, Ethiopia (*n* = 64976).

Variable	Category total	Discard	Non discard	Univariable analysis		Multivariable analysis	
				OR (95% CI)	*p*‐value	AOR (95% CI)	*p*‐value
Sex	Male	1952	39606	1		1	
	Female	1178	22240	0.93 (0.87–1.00)	0.006	0.95 (0.88–1.02)	0.17
Age group	18–27	1237	37857	1		1	
	28–37	940	12024	2.39 (2.19–2.61)	< 0.001	2.33 (2.13–2.55)	< 0.001
	≥ 38	953	11965	2.44 (2.24–2.66)	< 0.001	2.38 (2.18–2.60)	< 0.001
Donor type	Volunteer	3099	61648	1		1	
	Family	31	198	3.11 (2.13–4.56)	< 0.001	3.01 (2.06–4.41)	< 0.001
Collection site	Mobile	2693	53049				
	Center	437	8797	0.98 (0.88–1.08)	0.67	1.00 (0.90–1.11)	0.99
Name of blood bank	Dessie	1868	42443	1		1	
	Kemise‐	268	4090	1.49 (1.30–1.70)	< 0.001	1.47 (1.29–1.68)	< 0.001
	Woldia	709	11604	1.39 (1.27–1.52)	< 0.001	1.38 (1.26–1.51)	< 0.001
	Sekota	285	3709	1.75 (1.54–1.98)	< 0.001	1.73 (1.52–1.96)	< 0.001
Year	2022	888	17056	1		1	
	2023	832	17913	0.89 (0.81–0.98)	0.02	0.89 (0.81–0.98)	0.007
	2024	913	17485	1.00 (0.91–1.10)	0.95	1.00 (0.91–1.10)	0.96
	2025	497	9392	1.02 (0.91–1.14)	0.78	1.02 (0.91–1.14)	0.78
ABO group	O	1202	24614	1		1	
	A	1039	19189	1.06 (0.98–1.15)	0.16	1.03 (0.95–1.12)	0.17
	B	676	14884	0.93 (0.84–1.02)	0.13	0.93 (0.84–1.02)	0.13
	AB	213	3159	1.32 (1.14–1.53)	< 0.001	1.28 (1.10–1.49)	< 0.001
Rh factor	Rh Positive	2860	56256	1			
	Rh Negative	270	5590	0.95 (0.84–1.08)	0.43		

Abbreviations: AOR, adjusted odds ratio; CI, confidence interval; OR, unadjusted odds ratio.

In the study year; the odds of discard in 2023 were slightly lower (AOR = 0.89, 95% CI 0.81–0.98; *p *= 0.007), while 2024 and 2025 showed no significant difference compared with 2022 (AOR = 1.00, 95% CI 0.91–1.10; *p *= 0.96) and (AOR = 1.02, 95% CI 0.91–1.14; *p *= 0.78) respectively. Considering blood group; group AB had higher odds of discard (AOR = 1.28, 95% CI 1.10–1.49; *p *< 0.001) as compared with group O. Groups A (AOR = 1.03, 95% CI 0.95–1.12; *p *= 0.17) and B (AOR = 0.93, 95% CI 0.84–1.02; *p *= 0.13) showed no statistically significant differences. Rh negative units did not differ significantly from Rh positive units in discard odds (crude odds ratio (COR) = 0.95, 95% CI 0.84–1.08; *p *= 0.43). Sex of donors (AOR = 0.95, 95% CI 0.88–1.02; *p *= 0.17), and collection site (AOR = 1.00, 95% CI 0.90–1.11; *p *= 0.99) were not statistically significant difference as compared to the respective reference (Table 4).

#### 3.3.2 Associated Factors With Non‐TTI Related Blood Unit Discard

Factors associated with non‐TTI related blood unit discard were identified. Blood collected at fixed centers showed lower odds of non‐TTI discard as compared with blood collected in mobile sites (AOR = 0.65, 95% CI 0.57–0.74; *p *< 0.001). As compared with Dessie Blood Bank, Kemise and Sekota Blood Bank had higher odds of non‐TTI discard (AOR = 1.43, 95% CI 1.26–1.62; *p *< 0.001) and (AOR = 1.17, 95% CI 1.01–1.35; *p *= 0.03) respectively, while Woldia showed lower odds (AOR = 0.74, 95% CI 0.66–0.82; *p *< 0.001). Compared with the year 2022, the odds of non‐TTI discard in 2025 were significantly higher (AOR = 1.31, 95% CI 1.17–1.46; *p *< 0.001). Years 2023 (AOR = 0.90, 95% CI 0.80–0.99; *p *= 0.15) and 2024 (AOR = 0.11, 95% CI 0.91–1.11; *p *= 0.89) did not differ significantly. Regarding blood group, group A (AOR = 1.14, 95% CI 1.04–1.23; *p *= 0.001) and AB (AOR = 1.64, 95% CI 1.41–1.91; *p *< 0.001) had significantly higher adjusted odds of non ‐TTI discard when compared with group O. Group B showed no significant difference (AOR = 1.07, 95% CI 0.97–1.18; *p *= 0.30). Rh ‐negative units had substantially lower odds of non‐TTI discard compared with Rh ‐positive units (AOR = 0.36, 95% CI 0.28–0.44; *p *< 0.001) (Table 5).

**Table 5:** Logistic regression for factors associated with non‐TTI related blood unit discard in Eastern and Northeastern parts of the Amhara Regional State, Ethiopia (*n* = 64846).

Variable	Category total	Discard	Non discard	Univariable analysis		Multivariable analysis	
				OR (95% CI)	*p*‐value	AOR (95% CI)	*p*‐value
Sex	Male	1877	39606	1		1	
	Female	1123	22240	1.07 (0.99–1.15)	0.10	1.05 (0.97–1.14)	0.17
Age group	18–27	1893	37857	1		1	
	28–37	557	12024	0.93 (0.84–1.02)	0.13	0.90 (0.81–1.00)	0.14
	≥ 38	550	11965	0.92 (0.83–1.01)	0.09	0.91 (0.82–1.01)	0.08
Donor type	Volunteer	2991	61648	1			
	Family	9	198	0.94 (0.48–1.83)	0.85		
Collection site	Mobile	2713	52999	1		1	
	Center	287	8847	0.63 (0.56–0.72)	< 0.001	0.65 (0.57–0.74)	< 0.001
Name of blood bank	Dessie	2085	42443	1		1	
	Kemise	289	4090	1.44 (1.27–1.63)	< 0.001	1.43 (1.26–1.62)	< 0.001
	Woldia	411	11604	0.72 (0.65–0.80)	< 0.001	0.74 (0.66–0.82)	< 0.001
	Sekota	215	3709	1.18 (1.02–1.36)	0.02	1.17 (1.01–1.35)	0.03
Year	2022	803	17056	1		1	
	2023	778	17913	0.92 (0.83–1.02)	0.12	0.90 (0.80–0.99)	0.15
	2024	833	17485	1.01 (0.92–1.12)	0.81	1.01 (0.91–1.11)	0.89
	2025	586	9392	1.33 (1.19–1.48)	< 0.001	1.31 (1.17–1.46)	< 0.001
ABO group	O	1088	24564	1		1	
	A	989	19189	1.16 (1.06–1.27)	0.001	1.14(1.04–1.23)	0.001
	B	696	14934	1.05 (0.95–1.16)	0.32	1.07 (0.97–1.18)	0.30
	AB	227	3159	1.62 (1.39–1.89)	< 0.001	1.64 (1.41–1.91)	< 0.001
Rh factor	Rh Positive	2902	56216	1		1	
	Rh Negative	98	5630	0.34 (0.28–0.41)	< 0.001	0.36 (0.28–0.44)	< 0.001

Abbreviations: AOR, adjusted odds ratio; CI, confidence interval; OR, unadjusted odds ratio.

Sex of donors (AOR = 1.05, 95% CI 0.97–1.14; *p*= 0.17), age of the donors aged 28‐37 years (AOR = 0.90, 95% CI 0.81–1.00; *p *= 0.14) and ≥ 38 years (AOR = 0.91, 95% CI 0.82–1.01; *p *= 0.08) and donor type (COR = 0.94, 95% CI 0.48–1.83; *p *= 0.85) did not differ significantly from the respective reference group (Table 5).

## 4 Discussion

Our study's main goal was to assess the extent and reasons of TTIs and non‐TTI related blood and blood components discards in North Amhara, Ethiopia over a four ‐year period (2022–2025). Out of 67,976 blood units collected and prepared, 9.02% (6,130/67,976) were discarded. All Blood Banks had high discard rates; Kemise 12% (557/4647), Sekota 11.9% (500/4,209), Woldia 8.8% (1,120/12,724) and Dessie 8.5% (3,953/46,396). These high rate of waste presents a serious problem for a healthcare system that is already dealing with ongoing blood shortages. This finding indicates a significant loss of a scarce and expensive healthcare resource. These results showed that the necessity of context specific interventions as opposed to standard approaches used by all Blood Banks.

In our study the total discard rate was significantly higher than numbers reported in earlier Ethiopian studies carried out in Gondar [10], Dire Dawa [11], and other studies cared out another countries like Zimbabwe [12], Tanzania [2], Ghana [6], India [5, 13] and Central China [14]. The inclusion of non‐TTI‐related discard in this study can account for the discrepancy. However, our result was lower than previous studies in Burkina Faso [15], North Cameroon [16], and Uganda [17]. Variations in donor demographics, collection volume, storage capacity, staff experience, and blood consumption efficiency, failure to register blood unit discard in our study area, may account for these discrepancies.

Considering TTI ‐related discards, in all study period, accounting for 4.6% (3,130/67,976) units. HBV was the predominant driver 2% (1342/67,976) of infectious discard across all sites, reaching a peak of 4.1% (172/4,209) in Sekota, our result is consistent with data from sub‐Saharan Africa showing that HBV is still the most common cause of blood unit rejection during screening [4, 18, 19]. The persistent prevalence of HBV indicates that community transmission is still occurring and draws attention to deficiencies in preventive measures, such as the need for first ‐time donors and insufficient vaccination coverage.

The second and third most prevalent TTIs that contributed to blood disposal were syphilis 1.2% (814/67,976) and HIV 0.8% (543/67,976), respectively. For instance, syphilis prevalence was reaching a peak in Kemise 2.1% (106/4647). Similar trends have been seen in Ethiopia and nearby nations, where syphilis is still common among blood donors and reflects underlying socioeconomic and behavioral risk factors [10, 11]. Even though HIV‐related discard was less common than HBV and syphilis, its continued existence highlights the significance of upholding strict donor selection and screening protocols.

Regarding HCV it contributed to fewer discards 0.5% (372/67,976) but, it was consistently present throughout all research years, which is in line with regional data that indicate HCV prevalence among blood donors is lower but persistent [20]. The multiple infections represent units reactive to more than one infectious marker, which remained below 0.1% (59/67,976) across the study population.

In terms of time, TTI‐related discard was peaked at a year in 2024 and then marginally decreased in 2025. Changes in testing methods, donor recruitment strategies, could have an impact on these year to year variances. Syphilis prevalence was notably higher in Kemise 2.1% (106/4,647). TTI discard rate was also vary by Blood Banks, for instance Sekota Blood Bank demonstrated the highest prevalence of TTI ‐related discards 6.8% (285/4,209). These disparities might be due to challenges in donor screening, and donor management across Blood Banks.

Regarding to non‐TTI causes, out of the total 4.4% (3,000/67,976) discarded units. Expired blood units were the most common non‐TTI cause, which was in line with other studies [9, 13, 21]. Regarding, blood component discard PLT discard due to expiration was the most common 6.8% (269/13,984) which was in line with studies in Pakistan [21, 22] and followed by FFP and CRC which was similar in study in south India [21]. This is attributed to the short life time of PLT, mismatches between collection, demand, and dispatch systems, as well as possible weaknesses in stock management and inadequate facility to facility redistribution systems of blood units before their expiration. The second most frequent non‐TTI reason was small/underweight blood units, which suggests difficulties with donor screening and blood collection processes. Although they were less common, discards for discoloration, overweight units, and other reasons indicate handling and procedural problems that might be reduced with ongoing staff refreshment and adherence to standard operating procedures.

Regarding to Blood Banks kemise Blood Bank had recorded the most non ‐TTI discard rate 6.2% (289/4,649) followed by Sekota Blood Bank 5.1% (215/4,209), while Dessie and Woldia, Blood Banks were 4.5% (2,085/46,396) and 3.2% (411/12,724) respectively. These disparities might be due to challenges in inventory and collection management across Blood Banks. The overall discard trend showed for the first 3 years the annual prevalence of non ‐TTI discards was comparatively consistent 4.2% (803/18,973) in 2022, 4% (778/19,697) in 2023, and 4.5% (833/18,632) in 2024). However, in 2025, it sharply increased to 5.5% (586/10,674) mostly as a result of a rise in unit expiration. This change emphasizes that concurrent improvements in inventory management of blood and blood component is very essential.

Associated with factors causing TTI‐related blood and blood product discard; family/replacement donation was associated with a three‐fold higher odds of TTI related blood discard compared to voluntary donation. This finding aligns with the Ethiopian National Blood Bank Service's strategy of shifting toward 100% voluntary non ‐remunerated blood donation to minimize wastage and infection risks [4, 23]. Since replacement donors may sometimes, have less screening outcomes, less eligibility rates which may lead to higher deferral or post ‐collection discarding rates

Regarding donor age, compared to the 18–27 age group, the discard odds for donors aged 28–37 and ≥ 38 were 2.33 and 2.38 times higher for TTI‐related blood, respectively. This may reflect higher chance of exposure to TTI in older populations donors due to older individuals generally have had more lifetime partners, and medical history. Furthermore, significant variation in discard risk existed across Blood Bank sites; Kemise 1.47 times, Woldia 1.38 times, and Sekota 1.73 times showing higher adjusted odds than Dessie. These variations could be attributable to regional differences in TTI distribution and donor selection between Blood Banks.

Blood group AB was also associated with a 1.28‐fold higher odds of TTI‐related discard compared to group O. Literature suggests that ABO antigens may influence host‐pathogen interactions and infection susceptibility; specifically, group AB has been hypothesized to mirror blood‐group‐related susceptibility patterns reported for certain transfusion‐transmissible infections, including HIV [24, 25]. However, factors like sex, collection place, Rh factor, and study year were no significant correlation found for TTI‐related discard. This suggests that the presence of transfusion ‐transmitted diseases among screened donors, rather than facility‐level or demographic factors, was probably the primary determinant of TTI reject and similar findings have been reported in studies [26].

For non‐TTI‐related discards, significant statistical associations were observed with the collection site, blood bank name, collection period, and blood group. Blood collected at the main blood bank office or center demonstrated a 35% reduction in the odds of non‐TTI discard compared to mobile sites. This pattern is frequently linked to logistical challenges inherent to mobile campaigns, such as difficulties in maintaining a strict cold chain during transport, transit delays, and a heightened risk of mechanical issues like hemolysis [27].

In terms of specific facilities, Kemise and Sekota blood banks showed a 1.43 and 1.17‐fold increase in blood unit disposal for non‐TTI cases compared to Dessie, while Woldia demonstrated a 26% reduction. These discrepancies may stem from variations in infrastructure, where facilities experiencing greater logistical and storage constraints face higher non‐TTI losses, whereas those with optimized stock management and phlebotomy techniques achieve lower discard rates [28]. Additionally, while non‐TTI discards did not differ significantly between 2023 and 2024, the discard odds in 2025 were 1.31 times higher than in 2022. This trend coincided with a high volume of blood product expirations observed in 2025. Prior studies provide compelling evidence that inventory management and expiration dynamics heavily influence blood wastage patterns [29].

In the current cohort, Rh‐negative status was associated with a 64% lower odds of non‐TTI discard compared to Rh‐positive status. Conversely, blood groups A and AB carried a 1.14 and 1.64 times higher odds of non‐TTI discard compared to group O. Rather than reflecting intrinsic biological waste factors, this trend likely mirrors inventory dynamics and the high clinical demand for group O blood. Because Rh‐negative units are less common, they are typically managed and distributed with greater stringency, which may reduce the likelihood of wastage ‐ a finding consistent with similar reports [6, 30, 31, 32].

Finally, age, sex, and donor type did not significantly correlate with non‐TTI discard. This lack of association implies that minimizing non‐TTI wastage may depend more heavily on optimizing inventory control, storage, and distribution systems rather than targeting specific donor demographic groups.

## 5 Conclusion

Our study revealed that blood unit discard remains a significant challenge in Blood Bank services in eastern and northeastern Amhara, Ethiopia. The overall discard rate of 9.02% indicates a substantial loss of a valuable and limited healthcare resource. Both TTIs and non ‐TTIs causes contributed almost equally to blood unit wastage. HBV was identified as the most common infectious cause of discard, highlighting the continued burden of hepatitis B infection among blood donors.

Among non ‐infectious causes, blood unit expiration and underweight collection were the leading contributors, suggesting challenges in inventory management, donor screening, and blood collection practices. Additionally, blood collected during mobile campaigns was more likely to be non TTI related blood unit discarded compared to blood collected at fixed centers. The study also demonstrated that voluntary blood donation significantly reduces TTI related blood unit discard rates. Overall, improving inventory management systems, donor screening procedures, and blood collection practices is essential to minimize blood wastage and ensure efficient utilization of blood resources.

### 5.1 Limitations and Strength of the Study

The first limitation of our study was the nature of study design, retrospective, relying on previously recorded Blood Banks data, which may be subject to incomplete documentation of important variables like donor behavioral risk factors, educational status, and socioeconomic characteristics were not available in the Blood Bank records and therefore could not be analyzed. The other was component preparation which was performed only at Dessie Blood Bank, which may have influenced the distribution and analysis of blood component discard rates across the study facilities. Despite these limitations, the study provides valuable evidence regarding the magnitude, causes, and determinants of blood unit discard and can serve as an important reference for improving Blood Bank management and policy development in Ethiopia. In addition, our study is the first study which conducted blood unit discard due to both TTIs and non ‐TTIs causes in Ethiopia.

## Author Contributions

**Tesfaye Gessese:** methodology, writing – original draft, conceptualization, investigation, formal analysis, data curation, software. **Afewerk Habtamu Aseme:** investigation, writing – review and editing, methodology. **Zewudu Mulatie:** writing – review and editing, investigation, methodology. **Mikiyas Shemelis Achame:** writing – review and editing, investigation, methodology. **Sisay Desale:** investigation, methodology. **Saleamlak Sebsibe:** investigation, writing – review and editing, methodology. **Yonas Erkihun:** methodology, writing – review and editing, investigation. **Yeshimebet Kassa:** methodology, investigation, writing – review and editing. **Mahider Shimelis Feyisa:** investigation, writing – review and editing, methodology. **Mihreteab Alebachew:** supervision, validation, visualization. **Bruktawit Eshetu:** supervision, resources. **Tadesse Sisay:** supervision, visualization, validation.

## Funding

The authors have nothing to report.

## Ethics Statement

Ethical approval was obtained from the Institutional Review Board (IRB) of the College of Medicine and Health Sciences, Wollo University. We obtained permission letters from each Blood Bank prior to data collection. Written informed consent was not obtained from all participants since the data was retrospective.

## Conflicts of Interest

The authors declare no conflicts of interest.

## Transparency Statement

The corresponding author (**Tesfaye Gessese**) affirms that this manuscript is an honest, accurate, and transparent account of the study being reported; that no important aspects of the study have been omitted; and that any discrepancies from the study as planned have been explained. All authors have read and approved the final version of the manuscript. Mr. Tesfaye Gessese had full access to all of the data in this study and takes complete responsibility for the integrity of the data and the accuracy of the data analysis.

## Data Availability

The data that support the findings of this study are available from the corresponding author upon reasonable request. The datasets collected, used and analyzed are available from the corresponding author on reasonable request.
